# Formulation Design of Amiodarone Hydrochloride Tablets Optimized for a Continuous Manufacturing Process

**DOI:** 10.3390/pharmaceutics18070833

**Published:** 2026-07-07

**Authors:** Ju-Hyun Yoon, Chae-Won Jeon, Young-Joon Park, Joo-Eun Kim

**Affiliations:** 1 Department of Biopharmaceutical Chemistry, Kookmin University, Seoul 02707, Republic of Korea; wnguss2@naver.com (J.-H.Y.); great2k1@naver.com (C.-W.J.); 2 College of Pharmacy, Ajou University, 206 World Cup-Ro, Yeongtong-Gu, Suwon 16499, Republic of Korea; 3 Department of Biopharmaceutical Chemistry, School of Applied Chemistry, Kookmin University, Seoul 02707, Republic of Korea; 4 Department of Pharmaceutical Engineering, Kookmin University, Seoul 02707, Republic of Korea

**Keywords:** amiodarone hydrochloride, preformulation, continuous manufacturing, formulation, optimization

## Abstract

**Background:** Amiodarone hydrochloride is an antiarrhythmic agent, primarily classified as a Vaughan Williams Class III antiarrhythmic agent, used for the treatment and prevention of arrhythmia and designated as an essential national drug. Recently, the need to develop formulations suitable for continuous manufacturing, which is gaining significant attention in the pharmaceutical industry, has emerged. **Objective:** The objective of this study was to evaluate the formulation potential of amiodarone hydrochloride for the development of an oral tablet, with a specific focus on improving the initial dissolution rate to design a formulation optimized for continuous manufacturing. **Methods:** The primary physicochemical properties of amiodarone hydrochloride were predicted and subsequently validated through experimental characterization. Furthermore, the ratios of the binder and granulation liquid were optimized to facilitate robust and successful production via continuous manufacturing. **Results:** A new amiodarone hydrochloride tablet formulation, optimized for a continuous wet granulation process, was successfully developed by improving the initial dissolution rate through the optimization of the respective amounts of binder and granulation liquid. **Conclusions:** The optimal formulation design established in this research provides a critical foundation for enhancing the applicability of a continuous process in the manufacturing of amiodarone hydrochloride tablets.

## 1. Introduction

Arrhythmia refers to a group of conditions characterized by abnormalities in the normal electrophysiological activity of the heart, resulting in disturbances in cardiac rate and rhythm [1,2,3]. Pharmacotherapy plays a pivotal role in managing these irregular rhythms, and amiodarone hydrochloride (HCl) is considered one of the most widely used therapeutic agents due to its broad antiarrhythmic spectrum [4,5]. Amiodarone HCl is one of the antiarrhythmic drugs with the broadest therapeutic spectrum, effective against both supraventricular and ventricular arrhythmias [6,7]. Due to its essential role in improving survival rates in life-threatening situations such as acute cardiac arrest and the lack of viable alternatives, it has been designated as a national essential drug to ensure its stable supply is managed for disease control and public health [8,9]. The most widely used framework for understanding the pharmacological properties of antiarrhythmic drugs is the Vaughan Williams classification, proposed by Miles Vaughan Williams in 1970. This classification system categorizes drugs based on their electrophysiological mechanism of action into groups such as sodium channel blockers (Class I), beta-adrenergic blockers (Class II), potassium channel blockers (Class III), and calcium channel blockers (Class IV) [10]. According to this classification system, amiodarone HCl is primarily categorized as a Class III antiarrhythmic agent. Its principal mechanism of action involves the blockade of potassium (K^+^) channels in myocardial cells. This action delays the repolarization of the action potential, thereby prolonging the myocardial refractory period and suppressing arrhythmogenesis [11,12]. Interestingly, the pharmacological profile of amiodarone HCl is characterized by its multi-channel blocking properties; while it predominantly functions as a potent Class III agent, it also exhibits ancillary electrophysiological activities including sodium channel blockade (Class I), beta-adrenergic blockade (Class II), and calcium channel blockade (Class IV). However, its primary clinical and pharmacological designation remains strictly focused on its Class III antiarrhythmic profile [13,14,15,16].

The purpose of this study is to develop a formulation of amiodarone HCl tablets suitable for a continuous manufacturing (CM) process, enabling large-scale production. Pharmaceutical CM is a next-generation production paradigm wherein all unit operations, from raw material input to final product manufacturing, are integrated and performed continuously [17,18]. This represents a fundamental difference from the traditional batch process, where each operation is performed in discrete, sequential steps. A key advantage of CM is the implementation of real-time quality monitoring and control through process analytical technology (PAT). This enables the effective application of Quality by Design (QbD) principles, ensuring consistent product quality throughout the entire production process [19,20,21,22]. Furthermore, CM maximizes process efficiency through benefits such as a smaller equipment footprint, reduced production times, and decreased waste generation. Owing to these innovative advantages, CM shows high applicability, particularly in the production of high-value, low-volume pharmaceuticals and in new drug development requiring rapid market launch [23,24]. Recently, its scope of application has expanded throughout the industry, and it is poised to become the standard manufacturing technology in the future pharmaceutical sector [25,26,27].

In a preliminary study, when a formulation of amiodarone HCl tablets, which had demonstrated pharmaceutical equivalence in a batch process, was applied to a continuous process, a significant decrease in the initial dissolution rate was observed. The reduction was 20% in a simulated gastric environment (pH 1.2) and 30% in an intestinal environment (pH 4.0). This dissolution delay is scientifically attributed to the intense mechanical energy—specifically high shear stress and compressive forces—exerted by the twin screws during the continuous wet granulation process. This extensive energy induced rapid granule densification and a subsequent reduction in intra-granular porosity, which restricted capillary action and slowed the rate of water penetration into the tablet matrix. Therefore, the present study aimed to resolve this issue of reduced dissolution during process conversion and to develop a new, optimized formulation of amiodarone HCl tablets suitable for a continuous wet granulation process. To achieve this, the ratios of the binder and the granulation liquid—known to significantly impact the physical properties of granules and drug dissolution—were selected as key variables and systematically optimized under batch process conditions. The finally developed formulation was then evaluated for its process suitability and quality attributes to demonstrate its potential for efficient application in a continuous process.

## 2. Materials and Methods

### 2.1. Materials

The active pharmaceutical ingredient (API), amiodarone HCl, was purchased from Zhejiang Hengkang Pharmaceutical Co., Ltd. (Taizhou, Zhejiang, China). The excipients used were as follows: Pregelatinized starch 1500 was supplied by Colorcon Korea (Suwon, Gyeonggi-do, Republic of Korea), and lactose monohydrate 200M (Pharmatose 200M) was provided by DFE Pharma (Goch, North Rhine-Westphalia, Germany). Povidone K25 (PVP K25) was purchased from BASF (Ludwigshafen, Rhineland-Palatinate, Germany), while colloidal silicon dioxide (Aerosil 200) was obtained from Evonik (Essen, North Rhine-Westphalia, Germany). Magnesium stearate was supplied by Nitika Pharmaceutical Specialties Pvt. Ltd. (Maharashtra, Maharashtra, India). The solvents for analysis, acetonitrile and methanol, were purchased from Duksan Co., Ltd. (Ansan, Gyeonggi-do, Republic of Korea). All other reagents used were of analytical grade and were obtained from commercial sources.

### 2.2. Physicochemical Property Prediction of Amiodarone Hydrochloride

To minimize experimental trial-and-error and implement a rational, data-driven formulation design, a comprehensive physicochemical profile of amiodarone HCl was established using an in-house-developed artificial intelligence (AI)-based formulation design program. The program utilized the two-dimensional molecular structure of the API in the Simplified Molecular Input Line Entry System (SMILES) format as input, calculating 12 core physicochemical properties via embedded machine-learning-based prediction algorithms. These predictive parameters were systematically categorized to address various aspects of formulation development: (1) dissolution behavior within the gastrointestinal tract was characterized by evaluating pH-dependent solubility, aqueous solubility, and dissociation constants (pKa, pKb); (2) systemic absorption potential across biological membranes was estimated using lipophilicity and permeability indicators, including the partition coefficient (log P), pH-dependent distribution coefficient (log D), and Caco-2 cell permeability; (3) manufacturing stability was monitored through fundamental physico-chemical properties such as molecular weight, boiling point, and melting point; and (4) the overall success potential at the early development stage was assessed by integrating Lipinski’s “Rule of Five” compliance and bioavailability as indicators of druggability. This predictive model is currently hosted on a web-based platform, providing visualized and intuitive results through a graphical user interface (GUI), thereby enabling researchers to rapidly identify key API characteristics and effectively establish initial formulation strategies without the need for complex computational procedures.

### 2.3. Differential Scanning Calorimetry Analysis

The thermal properties and melting point of the API were analyzed using differential scanning calorimetry (DSC) to characterize its thermal behavior. Specifically, thermal profiles were monitored as indicators of sample purity, crystalline form, and potential stability changes during the manufacturing process. A DSC 200 (Hitachi Co., Ltd., Tokyo, Japan) was employed for the measurements. For sample preparation, approximately 9 mg of amiodarone HCl was accurately weighed using an electronic balance and hermetically sealed in a standard aluminum pan. An empty, identical aluminum pan served as the reference. To maintain an inert atmosphere, nitrogen gas (N_2_) was supplied at a flow rate of 50 mL/min throughout the analysis. The samples were initially stabilized under isothermal conditions at 30 °C for 5 min and subsequently heated from 30 °C to 200 °C at a linear heating rate of 10 °C/min.

### 2.4. Dynamic Light Scattering Analysis

The particle size characteristics of amiodarone HCl were analyzed in this study. Particle size and distribution were identified as key physical attributes associated with overall manufacturing consistency and bioavailability. To quantitatively evaluate these properties, a particle size analyzer (ELSZ-2000, Otsuka Electronics Korea Co., Ltd., Seongnam, Gyeonggi-do, Republic of Korea) based on the principle of dynamic light scattering (DLS) was utilized. For sample preparation, approximately 100 mg of amiodarone HCl was accurately weighed and dispersed in 50 mL of purified water to obtain a final concentration of 2 mg/mL. Intermittent vortex mixing was performed for 1 min to minimize particle agglomeration and achieve an appropriate concentration for measurement. To minimize potential measurement errors caused by the partial dissolution of the API in water, the sample analysis was conducted immediately within 5 min after the initial dispersion, ensuring that the suspension did not reach thermodynamic equilibrium solubility.

A 4.0 mL aliquot of the prepared suspension was carefully transferred into a disposable polymethyl methacrylate (PMMA) cuvette, with particular care taken to avoid the formation of air bubbles. To prevent measurement errors due to temperature fluctuations, the temperature of the cuvette holder was maintained at 25 °C during the analysis. To ensure reproducibility and reliability, all measurements were performed in triplicate, and the polydispersity index (PDI) was calculated as an indicator of the uniformity of the particle size distribution.

### 2.5. Solubility

In this study, both apparent solubility and equilibrium solubility tests were performed to comprehensively evaluate the solubility profile of amiodarone HCl.

Apparent solubility was determined to estimate the approximate solubility of the drug in various solvents. Briefly, 10 mg of amiodarone HCl was accurately weighed into a glass vial and stirred at 400 rpm using a magnetic bar. The test solvent was then added incrementally in 500 μL aliquots while visually monitoring the dissolution process. The end-point was defined as the moment when all particles completely disappeared, resulting in a clear solution. The total volume of solvent consumed until the end-point was recorded to calculate the solubility. If the drug remained undissolved even after the addition of 100 mL of solvent, it was classified as “practically insoluble” according to United States Pharmacopeia (USP) criteria.

Equilibrium solubility, representing the maximum concentration at which a drug is dissolved in a thermodynamically stable state under specific temperature and pH conditions, was measured to identify the intrinsic physicochemical properties of the drug. An excess amount of amiodarone HCl, exceeding the concentration identified in the apparent solubility test, was added to each solvent to prepare a supersaturated suspension. The suspensions were stirred at 400 rpm, and aliquots were sampled at 0 (initial), 2, 12, and 24 h. The quantitative analysis of these multi-time-point samples experimentally confirmed that the dissolved API concentration reached a constant plateau between 12 and 24 h, demonstrating that thermodynamic equilibrium solubility was successfully achieved within the 24 h timeframe. The collected samples were filtered through a 0.45 μm regenerated cellulose (RC) syringe filter to ensure the complete removal of undissolved particles. The resulting filtrate was diluted to a final concentration of approximately 0.1 mg/mL and quantified using high-performance liquid chromatography (HPLC). The equilibrium solubility was determined as the saturation concentration at which the measured values reached a plateau and no longer increased significantly over time.

### 2.6. Drug-Excipient Compatibility

To ensure the long-term stability and quality reliability of amiodarone HCl solid dosage forms, potential physicochemical interactions between the active API and various excipients were analyzed as a preliminary formulation step. Although the general properties of amiodarone HCl are well-documented, a significant literature gap exists regarding its specific impurity pathways—such as Impurity D formation—when exposed to various functional excipients under high-shear processing environments. To fill these missing data gaps from the previous literature, this study aimed to identify optimal excipient combinations that can withstand continuous manufacturing conditions by systematically monitoring drug degradation and the formation of related impurities that may occur during the formulation design process.

Twenty-one excipients were selected and precisely weighed at a 1:1 (*w*/*w*) ratio with amiodarone HCl to prepare binary mixtures. To ensure homogeneity, the mixtures were blended using a vortex mixer and subsequently sealed in amber glass vials to protect the samples from light and moisture. Following pharmaceutical stability guidelines, the prepared samples were stored for 4 weeks in stability chambers under room temperature (RT) conditions (25 ± 2 °C/60 ± 5% relative humidity (RH)) and accelerated conditions (AC) (40 ± 2 °C/75 ± 5% RH). The selected excipients included fillers (microcrystalline cellulose 101, lactose monohydrate 200M, mannitol SD 200, corn starch, and silicified microcrystalline cellulose 90), binders (pregelatinized starch 1500, povidone K25 and K90, hydroxypropyl cellulose-L, hydroxypropyl methylcellulose, and copovidone), and disintegrants (low-substituted hydroxypropyl cellulose, croscarmellose sodium, and sodium starch glycolate). Lubricants and glidants, such as magnesium stearate, sodium stearyl fumarate, and colloidal silicon dioxide (Aerosil 200), were also evaluated. Additionally, pH modifiers, including acidifying agents (citric acid and salicylic acid) and alkalinizing agents (magnesium carbonate and calcium hydroxide), were incorporated into the study. Samples were collected at initial, 2-week, and 4-week intervals to visually inspect for physical changes and to quantitatively assess chemical stability via HPLC. The HPLC analysis was utilized to monitor the formation of related impurities and to determine the individual and total impurity content. Through these assessments, the chemical interactions and degradation patterns over time were identified to finalize the compatibility profile of the API with each excipient.

### 2.7. Flowability Study

Powder flowability was evaluated as a critical control indicator to ensure the content uniformity of the final dosage form. All analyses were performed in accordance with the USP <1174> guidelines, where the bulk density and tapped density of the samples were precisely measured [28]. Initially, a specified mass of the sample was gently poured into a 100 mL graduated cylinder, and the bulk density was calculated based on the initial volume and mass of the powder. Subsequently, the cylinder was subjected to 100 taps, and the resulting tapped volume was recorded to determine the tapped density. Using the measured bulk and tapped density values, Carr’s index (CI) and the Hausner ratio (HR) were calculated according to Equations (1) and (2), respectively:

(1) $$Carr^{\prime }index\;\left(\%\right)=\;\frac{Tapped\;density-Bulk\;density}{Tapped\;density}\;\times 100$$

(2) $$Hausner\;ratio=\frac{Tapped\;density}{Bulk\;density}$$

The final flowability grade was assessed based on these calculated values in accordance with the established USP flowability scale.

### 2.8. Formulation Study of Amiodarone Hydrochloride

#### 2.8.1. Binder Ratio Study

A decrease in the initial dissolution rates across both gastric and intestinal pH environments was observed in amiodarone HCl tablets produced via CM. To ensure quality equivalence with the conventional batch process, formulation optimization research was essential to resolve this identified dissolution delay.

In this study, as a strategy to resolve the reduction in the initial dissolution rate occurring during the application of CM, research was conducted to optimize the ratio of the binder, which is a key factor determining the physical properties of granules in the wet granulation process. Povidone K25 was selected as the binder, and tablets were manufactured at four different levels of binder-to-total-formulation ratios: 5.71%, 2.86%, 2.00%, and 1.43% (Table 1). The manufacturing process involved granulating amiodarone HCl and excipients using a high-speed mixer (PM-C, PTK Co., Ltd., Gimpo, Gyeonggi-do, Republic of Korea), followed by compressing the resulting granules into tablets with consistent hardness and thickness using a rotary tablet press (PR-LM08, PTK Co., Ltd., Gimpo, Gyeonggi-do, Republic of Korea). Dissolution profiles and in-process control (IPC) tests were performed on each tablet batch to comparatively evaluate their quality attributes. Through this comparative analysis, the impact of binder ratio variations on the physical properties and drug release behavior of the tablets was analyzed in depth. In particular, by focusing on the dissolution patterns between the groups with the highest (5.71%) and lowest (1.43%) binder contents, this study aimed to derive the optimal binder ratio to effectively enhance the initial dissolution rate of amiodarone HCl tablets.

#### 2.8.2. Granulation Liquid Ratio Study

In this study, as part of a strategy to address the reduction in the initial dissolution rate observed during the implementation of the continuous process, research was conducted to optimize the amount of granulation liquid, a critical process variable that governs granule characteristics in the wet granulation process.

Purified water was employed as the granulation liquid, and tablets were manufactured at three different levels: 50 mg, 25 mg, and 20 mg per tablet (Table 1). The manufacturing process involved granulating amiodarone HCl and excipients using a high-speed mixer (PM-C, PTK Co., Ltd., Gimpo, Republic of Korea), followed by compressing the resulting granules into tablets with consistent hardness and thickness using a rotary tablet press (PR-LM08, PTK Co., Ltd., Gimpo, Republic of Korea). Dissolution profiles and IPC tests were performed for each tablet batch produced with varying amounts of granulation liquid to comparatively evaluate their quality attributes. Through this comparative analysis, the impact of variations in the granulation liquid volume on the physical properties and drug release behavior of the tablets was analyzed in depth. In particular, by focusing on the dissolution patterns between the groups with the highest (50 mg) and lowest (20 mg) liquid amounts, this study aimed to derive the optimal granulation liquid ratio to effectively enhance the initial dissolution rate of amiodarone HCl tablets.

#### 2.8.3. Filler Type Study

In this study, as part of a strategy to address the reduction in the initial dissolution rate observed during the implementation of the continuous process, research was conducted to optimize the types and particle sizes of the fillers used in the wet granulation process. Specifically, the impact of the particle size of lactose monohydrate, a widely used filler in tablet manufacturing, on the dissolution characteristics of amiodarone HCl was analyzed in depth.

To achieve this, tablets were manufactured using three different grades of lactose monohydrate with varying average particle sizes: Tablettose 80, SuperTab 11SD, and Pharmatose 200M (Table 2) [29,30]. The manufacturing process involved granulating amiodarone HCl and each filler using a high-speed mixer (PM-C, PTK Co., Ltd., Gimpo, Gyeonggi-do, Republic of Korea), followed by compressing the resulting granules into tablets with consistent physical properties using a rotary tablet press (PR-LM08, PTK Co., Ltd., Gimpo, Gyeonggi-do, Republic of Korea). Dissolution profiles and IPC tests were performed for each tablet batch to comparatively evaluate their quality attributes. Through this comparative analysis, the influence of filler particle size variations on the physical properties and drug release behavior of the tablets was investigated. In particular, by focusing on the dissolution patterns between tablets using the smallest and largest particle size grades of lactose monohydrate, this study aimed to derive the optimal filler type and particle size specifications to effectively enhance the initial dissolution rate of amiodarone HCl tablets.

#### 2.8.4. Filler Ratio Study

In this study, as a strategy to address the reduction in the initial dissolution rate, research was conducted to optimize the ratio of a key filler influencing tablet characteristics within the wet granulation process. Specifically, lactose monohydrate 200M (Pharmatose 200M) was selected as the target filler, and tablets were manufactured at three different levels: 38%, 27%, and 16% (*w*/*w*) relative to the total formulation (Table 1). The manufacturing process involved granulating the API and fillers using a high-speed mixer (PM-C, PTK Co., Ltd., Gimpo, Republic of Korea), followed by compressing the resulting granules into tablets with consistent physical properties using a rotary tablet press (PR-LM08, PTK Co., Ltd., Gimpo, Republic of Korea). Dissolution profiles and IPC tests were conducted for each tablet batch to comparatively evaluate their quality attributes. Through this comparative analysis, the impact of filler ratio variations on the physical properties and drug release behavior of the tablets was analyzed in depth. In particular, by focusing on the dissolution patterns between the groups with the highest (38%) and lowest (16%) excipient contents, this study aimed to derive the optimal filler ratio to effectively enhance the initial dissolution rate of amiodarone HCl tablets.

### 2.9. In-Process Control

To ensure the quality of the final dosage form, the flowability of the granules was evaluated during the manufacturing process, and key quality attributes of the tablets—including hardness, disintegration, and friability—were systematically measured.

The bulk density and tapped density of the granules were measured in accordance with the USP <1174> guidelines to comprehensively assess flowability via CI and the HR [31]. Briefly, a specified mass of granules was poured into a 100 mL graduated cylinder to determine the initial volume and calculate the bulk density. Subsequently, the cylinder was subjected to 100 taps, and the final volume was recorded once no further volume reduction was observed to determine the tapped density. The CI and HR were then calculated using the measured density values.

The hardness of ten tablets, randomly selected from each batch, was measured in kiloponds (kp) using a tablet hardness tester (YD-II, LABOAO, Zhengzhou, Henan, China). Results were expressed as the mean. Tablets with a hardness within the range of 4–7 kp were considered to meet the predefined quality specifications.

The disintegration time of amiodarone HCl tablets was determined according to USP <701> using a disintegration tester (BJ-2, Nanbei Instrument Ltd., Zhengzhou, China). Purified water maintained at 37.0 ± 2 °C served as the immersion medium [32]. Six tablets from each batch were placed in individual glass tubes and tested simultaneously. The time required for each of the six tablets to completely disintegrate and pass through the screen was recorded.

Friability was evaluated according to USP <1216> using a friability tester (CS-4, LABOAO, Zhengzhou, Henan, China) [31]. Twelve accurately pre-weighed tablets were placed in the drum and rotated at 25 rpm for 4 min (totaling 100 revolutions). After the rotation, the tablets were de-dusted and re-weighed to calculate the percentage weight loss. A weight loss not exceeding 1.0% was considered acceptable according to the compendial standards.

$$Fridability\;\left(\%\right)=\frac{Initial\;tablet\;weight-Final\;tablet\;weight}{Initial\;tablet\;weight}\times 100$$

### 2.10. Continuous Wet Granulation Process Study

**Figure 1:**
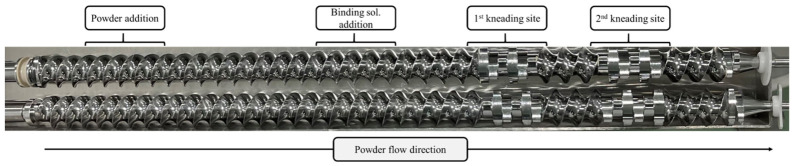
Twin-screw configuration used for the continuous manufacturing process.

The continuous wet granulation process in this study was performed using a twin-screw granulator (ConsiGma™-1, GEA, Bönen, North Rhine-Westphalia, Germany). The equipment is a die-plate-free, high-shear co-rotating twin-screw granulator with a length-to-diameter (L/D) ratio of 20:1, comprising a conveying section for powder transport and a mixing section for the intensive blending of the powder and purified water (Figure 1) [33,34].

The primary process parameters for the twin-screw granulator were established based on preliminary optimization screening and equipment capabilities to maintain a stable, continuous torque profile: a barrel temperature of 25 °C, a feed rate of 13.0 kg/h, and a screw speed of 700 rpm. The wet granules were automatically conveyed through the screw line to an integrated fluid bed dryer, where the drying process was completed at a drying temperature of 65 °C with an air volume of 70 m^3^/h. This drying temperature was selected on the basis of the API’s thermal stability profile determined by DSC (melting point initiation at 159.0 °C), ensuring rapid moisture removal without causing chemical degradation or phase transitions of amiodarone HCl. The loss on drying (LOD) of the final granules was precisely determined at 105 °C using a moisture analyzer (MB-120, OHAUS, Seoul, Republic of Korea).

### 2.11. Stability Study

Stability studies were conducted to assess the long-term quality stability of the finalized amiodarone HCl tablets and to verify their equivalence to the reference drug. The test and reference formulations were individually packaged in high-density polyethylene (HDPE) bottles, hermetically sealed, and placed in stability chambers. Stability testing was performed under two distinct storage conditions: RT (25 ± 2 °C/60 ± 5% RH) and AC (40 ± 2 °C/75 ± 5% RH). Samples were stored for a total of 6 months under each condition, with stability evaluations performed at the initial point (0, initial) and at 1, 3, and 6 months. At each sampling interval, the specimens were evaluated for appearance, drug content (assay), and related substances. This systematic stability testing aimed to scientifically demonstrate that the developed amiodarone HCl tablets maintain their quality standards and remain stable under the specified storage conditions over the intended period.

### 2.12. Dissolution Conditions

The in vitro dissolution test of the amiodarone HCl tablets was performed in accordance with the procedures outlined in the USP General Chapter <711> Dissolution, using a dissolution apparatus (708-DS Dissolution Apparatus, Agilent, Santa Clara, CA, USA) [35].

As amiodarone HCl is a biopharmaceutics classification system (BCS) Class II drug, characterized by low solubility and high permeability, the non-ionic surfactant polysorbate 80 (Tween 80) was added to the dissolution medium to ensure adequate solubility and maintain sink conditions, mimicking the in vivo environment. The test was conducted using 900 mL of either pH 1.2 or pH 4.0 buffer, each supplemented with 1% Tween 80, as the dissolution medium. The experimental conditions were maintained at a constant temperature of 37 ± 0.5 °C, with a paddle rotation speed of 75 rpm, for a total duration of 2 h. Aliquots (4 mL) were withdrawn from the dissolution vessel at a total of eight time points: 5, 10, 15, 30, 45, 60, 90, and 120 min. Each aliquot was immediately filtered through a 0.45 μm RC syringe filter to remove any undissolved particles. Finally, the filtered samples were quantitatively analyzed using the HPLC method detailed in Section 2.13.3, and the percentage of drug released over time was calculated.

$$Dissolution\;rate\;of\;a\;amiodarone\;hydrochloride\;\left(\%\right)=\frac{W_{S}\times A_{T}\times 9\times P}{A_{S}\times L}$$

A_T_ = Peak area of an amiodarone hydrochloride in the sample solution.

A_S_ = Peak area of an amiodarone hydrochloride in the standard solution.

W_S_ = Weight of amiodarone hydrochloride reference standard taken (mg).

9 = Dilution factor.

P = Purity of the reference standard (%).

L = Labeled amount of amiodarone hydrochloride per tablet (mg/tablet).

### 2.13. RP-HPLC Analysis

#### 2.13.1. Method for Quantitative Analysis

The content of amiodarone HCl was assayed using a HPLC method previously developed, optimized, and validated by our research group to improve peak symmetry and resolution [7]. The analysis was performed on an Agilent 1260 Infinity II HPLC system (Agilent Technologies, Santa Clara, CA, USA) equipped with an ultraviolet/visible (UV-Vis) detector. The separation was carried out on a Gemini C18 column (4.6 × 250 mm, 5 μm, 110 Å) with the column temperature maintained at 30 °C. The mobile phase was delivered at a flow rate of 1.0 mL/min, the injection volume was set to 10 μL, and the detection wavelength was 240 nm. The total run time for each analysis was 10 min. The analysis was conducted under isocratic conditions with a constant mobile phase composition. The mobile phase buffer was prepared by adding 3 mL of acetic acid and 20 mM of triethylamine to 1000 mL of purified water, with the final pH adjusted to 3.0 using acetic acid. The final mobile phase consisted of this prepared buffer and acetonitrile in a 30:70 (*v*/*v*) ratio. Under these HPLC conditions, the main peak for amiodarone HCl was observed at a retention time of 5.6 min.

#### 2.13.2. Analytical Method for Impurities in Amiodarone Hydrochloride

The related substances of amiodarone HCl were quantitatively analyzed using a HPLC system (Agilent 1260 Infinity II) equipped with an UV-Vis detector. To ensure regulatory compliance and baseline validity for impurity profiling, the analytical protocol was adapted from the official compendial method documented in the USP monographs for Amiodarone HCl Tablets. The chromatographic separation was performed on a Zorbax Eclipse XDB-C18 column (4.6 × 150 mm, 5 μm). The column temperature was maintained at 30 °C, and the mobile phase was delivered at a flow rate of 1.0 mL/min. The sample injection volume was 10 μL, the detection wavelength was set to 240 nm, and the total run time was 40 min. The analysis was conducted under isocratic conditions with a constant mobile phase composition. The buffer was prepared by mixing 3 mL of acetic acid with 1000 mL of purified water, with the final pH adjusted to 4.9 using ammonium hydroxide. The final mobile phase consisted of a mixture of acetonitrile, methanol, and the prepared buffer in a 40:30:30 (*v*/*v*/*v*) ratio. The diluent for sample preparation was a 50:50 (*v*/*v*) mixture of water and acetonitrile. Under these analytical conditions, the main peak for amiodarone HCl eluted at a retention time of approximately 24 min.

#### 2.13.3. Analytical Method for Dissolution

The dissolution analysis of amiodarone HCl was performed using a HPLC system equipped with an UV-Vis detector. This method was internally developed and optimized by our research team specifically for evaluating the drug release profiles under continuous manufacturing processing conditions. The chromatographic separation was achieved on a Zorbax Eclipse Plus C18 column (4.6 × 150 mm, 5 μm), (Agilent Technologies, Santa Clara, CA, USA) with the column temperature maintained at 40 °C. The mobile phase was delivered at a flow rate of 1.0 mL/min, the injection volume was 10 μL, and the detection wavelength was set to 240 nm. The total run time for each analysis was 7 min. The analysis was conducted under isocratic conditions with a constant mobile phase composition. First, Solution A was prepared by mixing 5 mL of triethylamine with 1000 mL of purified water. The final mobile phase consisted of a mixture of acetonitrile, methanol, and Solution A in a 42:38:20 (*v*/*v*/*v*) ratio, with the final pH adjusted to 4.0 using phosphoric acid.

## 3. Results and Discussion

### 3.1. Physicochemical Property of Amiodarone Hydrochloride

In this study, the core characteristics of amiodarone HCl were analyzed to establish a scientific rationale for developing an effective formulation strategy. The pKa of amiodarone HCl was 9.08, identifying it as a weakly basic drug according to established literature sources, which implies that its solubility varies significantly depending on the pH fluctuations within the gastrointestinal tract [36]. In highly acidic environments such as the stomach (pH 1–3), the drug molecules exist predominantly in an ionized form, potentially leading to relatively higher solubility. However, as the drug moves through the duodenum into the small intestine, where the pH approaches neutrality (pH > 6.8), the proportion of the unionized form increases sharply, resulting in a marked decrease in solubility. Given that a drug must be in a dissolved state to be absorbed, the low solubility in the small intestine—the primary site of absorption—represents a major barrier to oral bioavailability. Considering both this low solubility and the predicted high membrane permeability, amiodarone HCl can be classified as a BCS Class II drug according to the BCS. The predicted aqueous solubility of 0.00476 mg/mL further supports this classification (Table 3). Consequently, the most critical challenge in the formulation development of amiodarone HCl is the effective enhancement of its solubility and dissolution rate within the intestinal environment. Therefore, dissolution testing will serve as a critical quality attribute (CQA) for evaluating the quality of the newly developed tablets and predicting their in vivo performance. These findings provide a strategic direction for formulation design, such as particle size reduction or the incorporation of surfactants, to improve the overall dissolution profile.

### 3.2. Comparison Between AI Predictions and Experimental Results

**Figure 2:**
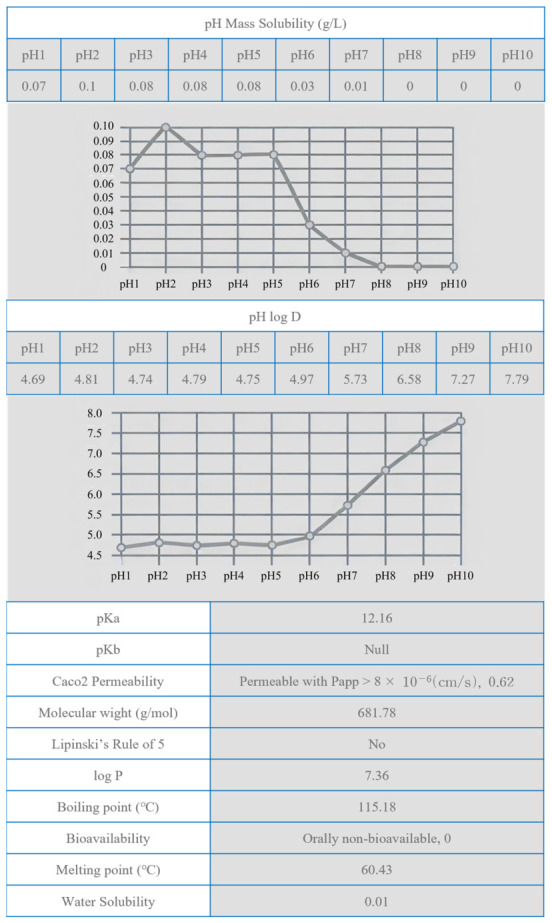
Prediction results of physicochemical properties using an AI program.

The success of pharmaceutical formulation development is predicated on an accurate and in-depth understanding of the intrinsic physicochemical properties of the API. In this study, an in-house-developed AI-based formulation design program was utilized to analyze the core attributes of amiodarone HCl. By inputting the chemical structure in the SMILES format, the program successfully predicted 12 key physicochemical parameters, including solubility, pKa, log P, melting point, and boiling point, via embedded machine learning algorithms. Notably, all predictive profiles and parameters generated by the AI program in Figure 2 and Table 4 are strictly valid for the amiodarone HCl salt form, as the input structure explicitly incorporated the hydrochloride counter-ion. These predicted data points are not merely a collection of individual information but serve as a comprehensive basis for forecasting the in vivo behavior and manufacturability of the drug. Ensuring the reliability of predicted data is essential for the practical application of in silico models. Therefore, the accuracy of the model was verified by comparing the predicted values with experimental values reported in existing literature. The analysis revealed a high concordance rate of 76.3% between the predicted and experimental values, statistically confirming that the AI program accurately captures the complex characteristics of amiodarone HCl. While the AI program demonstrated an overall acceptable concordance rate, a scientifically rigorous evaluation of Table 4 reveals significant numerical discrepancies in specific thermodynamic parameters, particularly the melting point and boiling point. The observed numerical discrepancies in certain properties, such as the predicted melting point (60.43 °C) versus the actual experimental data (156 °C), are attributed to the inherent mathematical limitations of the machine learning algorithm in fully calculating ionic crystal lattice energies. These pronounced differences highlight the inherent limitations of the current in silico machine learning model. The embedded algorithms primarily utilize structural descriptors that excel at estimating intramolecular properties and phase-partitioning characteristics, such as pKa and log P, which showed excellent agreement with experimental data. However, they possess a recognized systemic limitation in accurately calculating macroscopic thermodynamic transitions that depend heavily on intermolecular crystal lattice constraints. For amiodarone HCl, the intense electrostatic interactions and packing energy introduced by the hydrochloride counter-ion in its solid crystalline state were underrepresented by the model, leading to a substantial underestimation of thermal values. Acknowledging these specific model boundaries is essential; while the AI tool provides rapid, high-throughput screening utility for molecular-level behaviors like solubility and ionization, its quantitative predictions for solid-state thermal properties require careful experimental verification and further optimization of the algorithmic training matrix.

Such AI-based in silico screening is of significant value as it enables the rapid identification of properties that critically impact drug quality and allows for the establishment of management strategies prior to performing actual experiments. In conclusion, the AI predictive model employed in this study will serve as a vital foundation for reducing trial-and-error in subsequent development phases and enabling rational formulation design based on robust scientific evidence.

### 3.3. Differential Scanning Calorimetry Results

**Figure 3:**
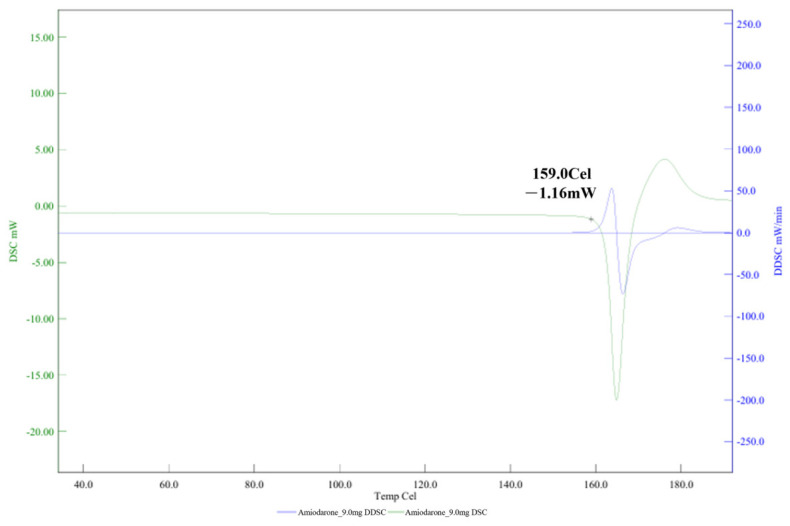
Melting point analysis of amiodarone hydrochloride.

The thermal properties of amiodarone HCl were precisely characterized using DSC. The analysis revealed a single, distinct, and symmetrical endothermic peak, which initiated at 159.0 °C and concluded at 168.9 °C. This peak corresponds to the melting process, during which the absorbed thermal energy disrupts the crystal lattice, facilitating the phase transition from solid to liquid. The sharp and singular nature of this peak strongly suggests that the amiodarone HCl used in this study is a high-purity crystalline substance with negligible impurities. Typically, significant impurities or the coexistence of amorphous forms leads to melting point depression, resulting in broad and asymmetrical peak profiles (Figure 3).

In this study, the melting point (T_m_) was determined as the extrapolated onset temperature where the phase transition of drug molecules initiates, which was measured at 159.0 °C This relatively high melting point suggests that amiodarone HCl can maintain a physically stable solid state against the heat typically generated during general formulation processes. Consequently, these DSC results provide critical scientific evidence that amiodarone HCl possesses the excellent thermal stability and high-purity crystallinity required for successful formulation development.

### 3.4. Dynamic Light Scattering Results

**Figure 4:**
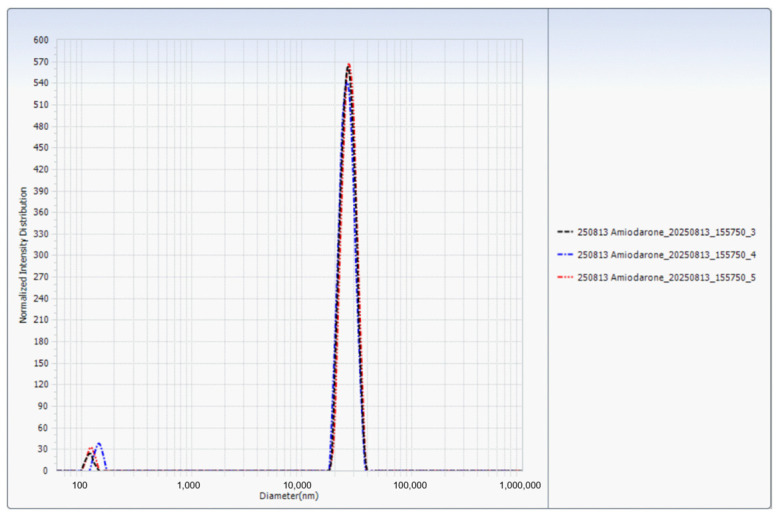
Particle size distribution of amiodarone hydrochloride.

In this study, the particle size characteristics of the amiodarone HCl raw material were precisely analyzed using DLS. According to the results obtained from triplicate measurements, the volume-based cumulative particle size distribution values—D_10_, D_50_, and D_90_—were determined to be 20.3 μm, 25.0 μm, and 30.6 μm, respectively (Figure 4). Notably, the PDI, an indicator of the uniformity of the particle size distribution, exhibited an exceptionally high range, spanning from 4.261 to 5.159 (Table 5). Generally, in DLS analysis, a monodisperse sample typically shows a PDI value of less than 0.1, whereas values exceeding 1.0 are considered to represent an extremely broad distribution. Therefore, the observed PDI values clearly demonstrate that the amiodarone HCl possesses a broad and heterogeneous particle size distribution. These findings strongly suggest that rather than utilizing the raw amiodarone HCl in its current state, essential pre-processing steps—such as reducing the particle size and controlling the distribution through milling or micronization, or intentionally increasing and homogenizing the size through processes like wet granulation—must be implemented prior to the formulation process.

### 3.5. Evaluation of Solubility

**Figure 5:**
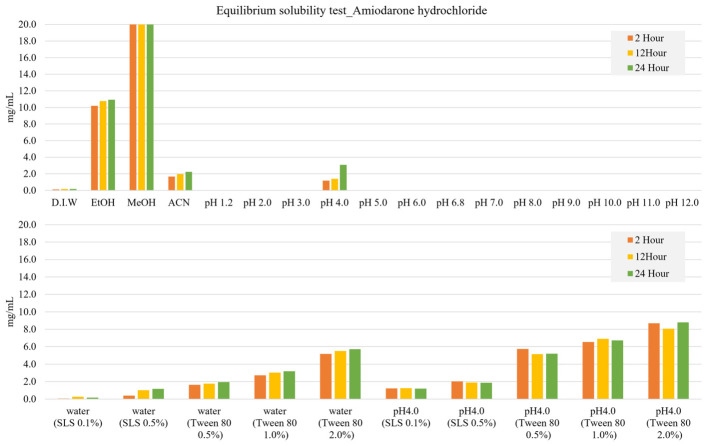
Evaluation of equilibrium solubility of amiodarone hydrochloride according to solvent type.

A comprehensive evaluation of the solubility profile of amiodarone HCl revealed that it is classified as “practically insoluble” or “very slightly soluble” in most solvents according to the USP solubility criteria. While showing a tendency to dissolve in organic solvents such as methanol and ethanol, the drug exhibited overall very low solubility in aqueous solutions and various pH buffers. Specifically, pH-dependent assessments showed that the drug is slightly soluble at pH 4.0, while remaining essentially insoluble in neutral and basic pH ranges. This low solubility is a major factor limiting the bioavailability of amiodarone HCl, a BCS Class II drug. To address this, sodium lauryl sulfate (SLS), an anionic surfactant, and Tween 80, a non-ionic surfactant, were incorporated to observe their effects on solubility enhancement. Both surfactants improved solubility, but Tween 80 demonstrated a superior enhancement effect compared to SLS (Figure 5).

Quantitative verification via equilibrium solubility testing showed that solubility in pure pH 4.0 buffer was 3.06 mg/mL after 24 h. In contrast, in pH 4.0 buffer containing 1% Tween 80, the solubility reached 6.54 mg/mL within 2 h and maintained a stable level of 6.71 mg/mL after 24 h, representing a more than twofold increase (Table 6). These results provide critical insights for the design of in vitro dissolution testing for amiodarone HCl. To accurately evaluate drug release patterns, sink conditions—where the drug concentration is maintained at less than 30% of its saturation solubility—must be met. Consequently, pH 4.0 buffer containing 1% Tween 80 is considered an appropriate dissolution medium to effectively satisfy sink conditions, thereby ensuring the acquisition of reliable data for predicting in vivo drug behavior.

### 3.6. Evaluation of Drug-Excipient Compatibility

In this study, the physical and chemical stability of binary mixtures consisting of amiodarone HCl and 21 types of excipients in a 1:1 (*w*/*w*) ratio were comprehensively evaluated over a 4-week storage period. The selection of these twenty-one excipients was purposely planned to focus on defining functional formulation boundaries and literature gaps rather than merely replicating standard compendial data from known commercial formulations. In particular, organic acids (citric acid and salicylic acid) were incorporated to evaluate their potential as microenvironmental pH modifiers aimed at enhancing the dissolution of the weakly basic amiodarone HCl in higher pH environments. Conversely, basic agents (magnesium carbonate and calcium hydroxide) were intentionally introduced into the matrix as structural stress challenges to systematically elucidate the chemical degradation pathways and mechanisms of impurity formation under localized alkaline microenvironments.

Visual inspections of all mixtures throughout the storage duration revealed no significant changes in physical appearance—such as discoloration, agglomeration, or hygroscopicity—compared to their initial states. These observations suggest that amiodarone HCl maintains a physically stable state with most of the tested excipients under the specified conditions. Furthermore, HPLC analysis was performed to quantitatively evaluate the formation of related substances resulting from potential chemical interactions. The stability acceptance criteria were established as ≤0.5% for “Impurity D” (a specific individual impurity) and ≤1.0% for the “total impurities.”

The analytical results indicated that 18 out of the 21 excipients satisfied these stability criteria under both RT and AC for the entire duration. However, three excipients—sodium stearyl fumarate, magnesium carbonate, and calcium hydroxide—exhibited incompatibility with the API by producing impurities that exceeded the established limits (Table 7). Specifically, the lubricant sodium stearyl fumarate showed an Impurity D level exceeding the 0.5% threshold after 2 weeks of storage under AC. Moreover, for the alkalinizing agents magnesium carbonate and calcium hydroxide, both Impurity D and total impurity levels significantly surpassed the criteria at both the 2-week and 4-week intervals under AC. The pronounced formation of impurities in mixtures with basic compounds strongly suggests that amiodarone HCl may undergo accelerated degradation when exposed to alkaline environments. This is a critical factor that must be addressed during formulation design to ensure long-term stability.

In conclusion, this compatibility study confirmed that mixtures appearing physically stable may still be chemically incompatible, leading to the successful identification of specific incompatible excipients. Consequently, these three substances will be excluded from the final formulation, which will instead prioritize the use of excipients with verified stability to ensure a robust and stable product.

### 3.7. Evaluation of Flowability

In this study, the flowability of amiodarone HCl was quantitatively evaluated by measuring CI and the HR, which are compressibility indices specified in the USP. The measured bulk density and tapped density were 0.4950 g/mL and 0.7071 g/mL, respectively. Based on these values, the calculated CI was 30% and the HR was 1.43, classifying the material under the “Poor” flowability grade according to standard pharmacopeial criteria (Table 8). Such high CI and HR values indicate that amiodarone HCl powder possesses strong inter-particulate cohesive forces and high internal friction, leading to significant agglomeration and resistance to free flow. Since this poor flowability can cause critical issues—such as inconsistent die filling and weight variation—during large-scale tablet manufacturing, optimization is essential. To fundamentally address these limitations, two primary strategies were established. First, wet granulation was employed to transform fine API particles into larger, denser, and more spherical granules using a binder, thereby inherently improving flow characteristics. Second, a glidant, such as colloidal silicon dioxide, was incorporated into the formulation to coat the granule surfaces. This approach aims to maximize flowability by reducing inter-particulate friction and neutralizing electrostatic attractions. Through these integrated strategies, the study aimed to overcome the inherent flowability limitations of amiodarone HCl and develop a robust formulation suitable for stable and reproducible tablet production.

### 3.8. Results of Amiodarone Hydrochloride Formulation Development

#### 3.8.1. Results of Binder Ratio Study

As part of the formulation optimization for amiodarone HCl tablets, in vitro dissolution studies were conducted to enhance the initial dissolution rate in both gastric and intestinal pH environments. Dissolution was evaluated in pH 1.2 and pH 4.0 buffers containing 1% Tween 80, and equivalence was assessed by calculating the similarity factor (*f*_2_) according to USP guidelines.

**Figure 6:**
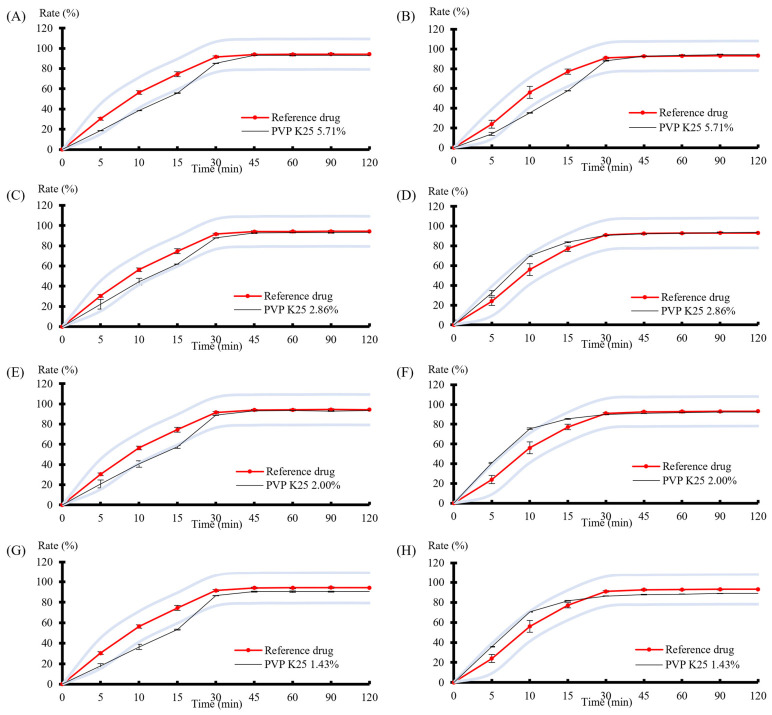
In vitro drug release from test formulations with varying binder ratios: (**A**) 5.71% povidone K25 in pH 4.0 medium; (**B**) 5.71% povidone K25 in pH 1.2 medium; (**C**) 2.86% povidone K25 in pH 4.0 medium; (**D**) 2.86% povidone K25 in pH 1.2 medium; (**E**) 2.00% povidone K25 in pH 4.0 medium; (**F**) 2.00% povidone K25 in pH 1.2 medium; (**G**) 1.43% povidone K25 in pH 4.0 medium; (**H**) 1.43% povidone K25 in pH 1.2 medium.

The study focused on the ratio of the binder, Povidone K25, as a key factor influencing the initial dissolution rate, and tablets manufactured with four different ratios were comparatively evaluated. Interestingly, a trend contrary to the general expectation—that a decrease in the binder ratio would proportionally increase the initial dissolution rate—was observed. Instead, the formulation where the binder ratio was reduced from the initial 5.71% to 2.86% exhibited the most significant increase in the initial dissolution rate in both pH environments (Figure 6). This is consistent with the similarity factor results against the reference drug, which yielded *f*_2_ values of 58.2 at pH 1.2 and 61.4 at pH 4.0, satisfying the acceptance criterion (*f*_2_ ≥ 50) and representing the most superior outcome.

Furthermore, IPC studies demonstrated that granules prepared with 2.86% binder exhibited excellent flowability, with a CI of less than 20%. The resulting tablets also showed favorable physical properties, including a rapid disintegration time of 3.43 ± 0.4 min and a very low friability of 0.29 ± 0.1% (Table 9).

Comprehensive consideration of both the dissolution and IPC results indicated that the formulation utilizing 2.86% Povidone K25 provided the optimal balance between the target enhancement of initial dissolution and the required physical attributes. Consequently, the binder ratio was finalized at 2.86%, providing the foundation for subsequent formulation optimization studies.

#### 3.8.2. Results of Granulation Liquid Ratio Study

**Figure 7:**
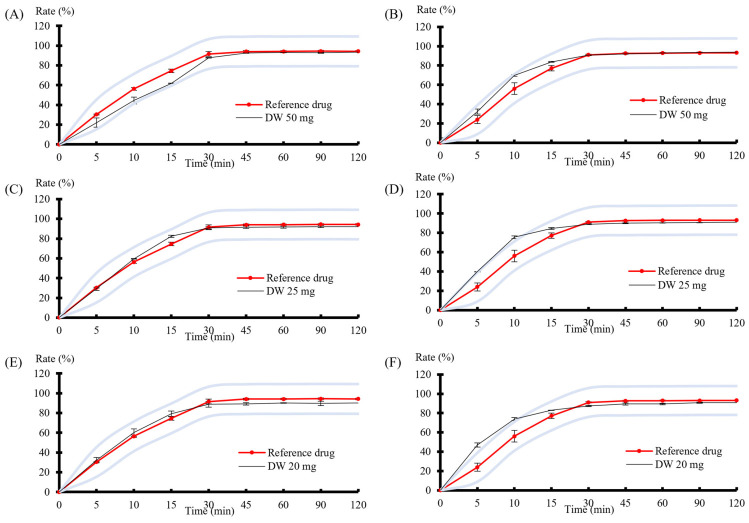
In vitro drug release from test formulations with varying granulation liquid ratios: (**A**) 50 mg DW in pH 4.0 medium; (**B**) 50 mg DW in pH 1.2 medium; (**C**) 25 mg DW in pH 4.0 medium; (**D**) 25 mg DW in pH 1.2 medium; (**E**) 20 mg DW in pH 4.0 medium; (**F**) 20 mg DW in pH 1.2 medium.

In this study, the impact of the granulation liquid amount on the initial dissolution rate was evaluated by comparing tablets manufactured at three different levels. The results demonstrated a clear trend: as the amount of granulation liquid decreased, the initial dissolution rate improved. However, it was also observed that an excessive reduction in the liquid volume led to process limitations, specifically preventing proper granule formation. Notably, the formulation in which the granulation liquid was reduced from 50 mg to 25 mg per tablet exhibited the most significant enhancement in the initial dissolution rate in both gastric and intestinal pH environments (Figure 7). This improvement was consistent with the similarity factor results against the reference drug, yielding f_2_ values of 54.4 at pH 1.2 and 70.9 at pH 4.0, thereby satisfying the equivalence criterion (*f*_2_ ≥ 50) and representing the most superior outcome.

Furthermore, additional IPC evaluations revealed that this specific formulation possessed exceptional physical properties. The granules exhibited excellent flowability, with a CI of less than 10%. The resulting tablets also demonstrated a very rapid disintegration time of 1.66 ± 0.2 min and a low friability of 0.29 ± 0.1%, confirming that they met the requirements for both mechanical strength and rapid drug release (Table 10).

Comprehensive assessment of the dissolution and IPC results confirmed that the formulation with the granulation liquid amount adjusted to 25 mg per tablet was the optimal design, satisfying both the target initial dissolution enhancement and the necessary physical attributes. Consequently, the amount of granulation liquid for the final formulation of the amiodarone HCl tablets was finalized at 25 mg per tablet.

#### 3.8.3. Results of Filler Type Study

In this study, the impact of the particle size of the filler (lactose monohydrate) on the initial dissolution rate was evaluated by comparing tablets manufactured with three different grades of lactose monohydrate. Contrary to the general expectation that larger filler particles would enhance the initial dissolution rate, no proportional increase in dissolution was observed with increasing particle size. In fact, the use of larger-sized lactose grades led to a reduction in the dissolution rate at the 15 and 30 min intervals.

**Figure 8:**
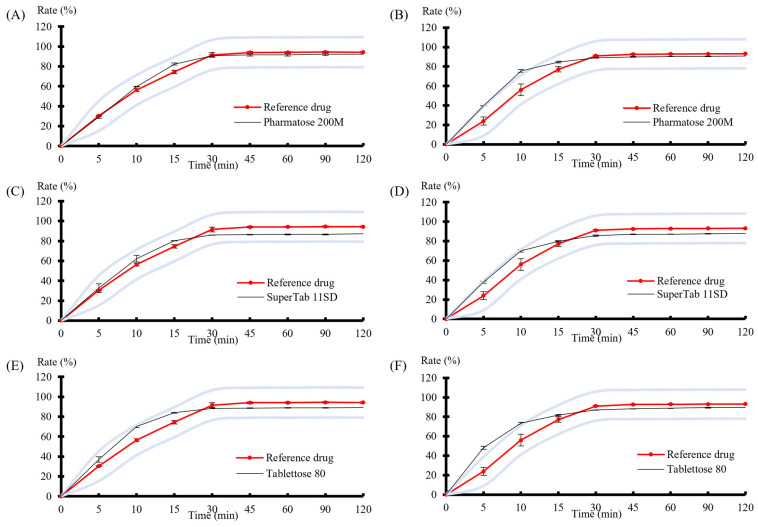
In vitro drug release from test formulations with varying filler types: (**A**) Pharmatose 200M in pH 4.0 medium; (**B**) Pharmatose 200M in pH 1.2 medium; (**C**) SuperTab 11SD in pH 4.0 medium; (**D**) SuperTab 11SD in pH 1.2 medium; (**E**) Tablettose 80 in pH 4.0 medium; (**F**) Tablettose 80 in pH 1.2 medium.

Specifically, among the three grades tested, the tablets formulated with Pharmatose 200M—which was used in the original formulation—demonstrated the most significant initial dissolution rate in both gastric and intestinal pH environments (Figure 8). This is consistent with the similarity factor results against the reference drug, which yielded values of 54.4 at pH 1.2 and 70.9 at pH 4.0, thereby satisfying the equivalence criterion (*f*_2_ ≥ 50) and representing the most superior performance.

Furthermore, an additional IPC evaluation of the formulation using Pharmatose 200M revealed exceptional physical properties. The granules exhibited excellent flowability, with a CI of less than 10%. Moreover, the resulting tablets showed a very rapid disintegration time of 1.66 ± 0.2 min and a low friability of 0.29 ± 0.1%, confirming that the formulation met the requirements for both mechanical strength and rapid drug release (Table 11).

Comprehensive assessment of the dissolution and IPC results proved that Pharmatose 200M is the optimal excipient, satisfying both the initial dissolution rate and process suitability. Consequently, Pharmatose 200M was confirmed as the excipient for this amiodarone HCl formulation study, and subsequent research was conducted to determine the optimal ratio of this excipient.

#### 3.8.4. Results of Filler Ratio Study

In this study, the impact of the filler (lactose monohydrate) ratio on the initial dissolution rate was evaluated by comparing tablets manufactured at three different levels. Analytical results revealed that the formulation with a 38% lactose monohydrate ratio exhibited a significant decrease in the initial dissolution rate at pH 4.0. This suggests that an excessive amount of the filler may have exerted complex effects on the granule structure and disintegration characteristics, potentially hindering drug release.

**Figure 9:**
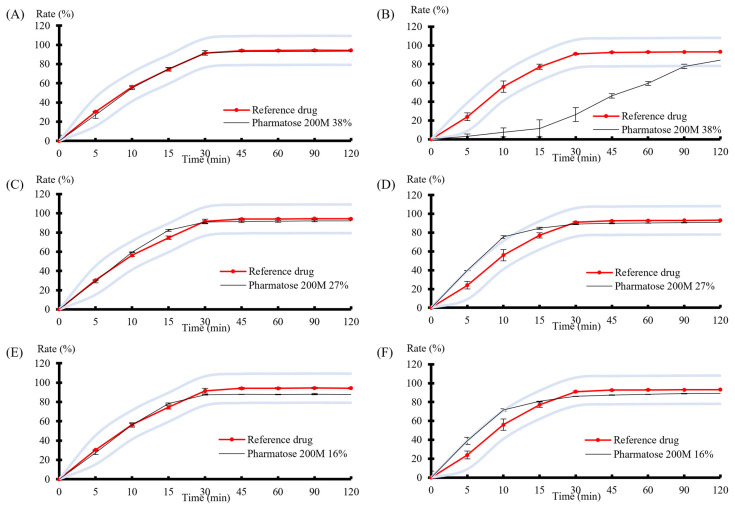
In vitro drug release from test formulations with varying filler ratios: (**A**) 38% Pharmatose 200M in pH 4.0 medium; (**B**) 38% Pharmatose 200M in pH 1.2 medium; (**C**) 27% Pharmatose 200M in pH 4.0 medium; (**D**) 27% Pharmatose 200M in pH 1.2 medium; (**E**) 16% Pharmatose 200M in pH 4.0 medium; (**F**) 16% Pharmatose 200M in pH 1.2 medium.

Conversely, the 27% ratio—representing the original formulation—demonstrated the most significant initial dissolution rate in both gastric and intestinal pH environments (Figure 9). This result aligns with the similarity factor values against the reference drug, which were 54.4 at pH 1.2 and 70.9 at pH 4.0, thereby satisfying the equivalence criterion (*f*_2_ ≥ 50) and representing the most superior performance.

Furthermore, an additional IPC evaluation of the 27% lactose monohydrate formulation revealed exceptional physical properties. The granules showed excellent flowability with a CI of less than 10%, and the resulting tablets exhibited a very rapid disintegration time of 1.66 ± 0.2 min along with a low friability of 0.29 ± 0.1% (Table 12).

Comprehensive assessment of the dissolution and IPC results proved that the formulation containing 27% lactose monohydrate is the optimal design, satisfying both the initial dissolution rate and process suitability. Consequently, the final ratio of the excipient for this amiodarone HCl formulation was confirmed at 27% (Table 13).

#### 3.8.5. Establishment of QbD Framework: Correlation Between Critical Process Parameters (CPPs) and CQAs

To establish a scientifically rigorous QbD framework for the continuous manufacturing of amiodarone HCl tablets, the CQAs and CPPs were clearly defined and systematically correlated. The target CQAs of the final dosage form were established as the initial dissolution rate (specifically aiming for rapid drug release within 15 min under both gastric and intestinal environments to overcome processing constraints), disintegration time, and tablet hardness to ensure pharmaceutical equivalence to the reference drug. Concurrently, the CPPs governing these quality attributes within the continuous twin-screw wet granulation line were identified as the binder ratio, granulation liquid ratio, and mechanical engineering variables (feed rate and screw speed). These CPPs critically dictate the raw powder agglomeration behavior; variations in the liquid-to-solid ratios or screw shear stress directly alter the internal porosity and density of the resulting granules, which subsequently act as the underlying mechanisms governing the final tablet CQAs, particularly the water penetration rate during disintegration and initial drug dissolution kinetics.

To deeply contextualize this work within the current state of the art of pharmaceutical CM, it is crucial to position our engineering approach against the broader paradigm shifts in twin-screw wet granulation. Recent milestone studies have extensively focused on scale-independent parameters such as Specific Mechanical Energy (SME) and Residence Time Distribution (RTD) to predict CQAs from process variables [34]. Furthermore, modern continuous frameworks heavily lean toward de novo formulation designs engineered exclusively for high-throughput lines. However, a significant knowledge and practical gap remains regarding the seamless operational transition of highly regulatory-sensitive, low-solubility drugs like amiodarone HCl from established batch matrices to continuous platforms. Our previous research successfully mapped out a robust continuous design space using a central composite design by isolating twin-screw speeds and milling parameters. Building upon these cumulative advancements, the present study establishes a unique state-of-the-art position by developing a descriptive QbD optimization strategy that specifically counteracts continuous granulation-induced densification and subsequent dissolution delays without altering the core qualitative excipient composition of the legacy batch formulation. This direct-transfer formulation approach minimizes regulatory friction and manufacturing capital burdens, serving as a pivotal technological reference for modernizing the manufacturing lines of BCS Class II drugs.

### 3.9. Comparative Analysis of Quality Equivalence: Batch vs. Continuous Manufacturing

**Figure 10:**
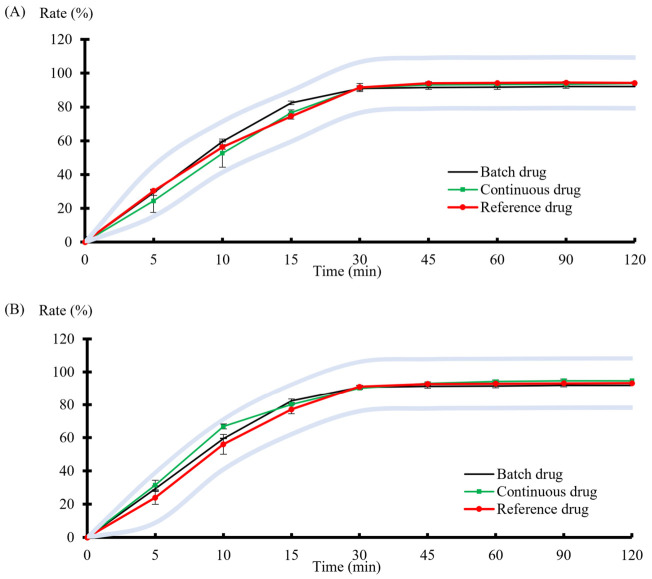
Comparison of dissolution profiles between tablets manufactured via batch and continuous processes: (**A**) pH 4.0 medium, (**B**) pH 1.2 medium.

A critical and mechanistic interpretation of the comparative data reveals a subtle yet scientifically significant structural divergence between the two manufacturing platforms. While the batch-processed tablets achieved a rapid mean disintegration time of 1.66 ± 0.2 min, the continuous-processed tablets exhibited a prolonged disintegration average of 6.41 ± 1.9 min (Table 14). Rather than a superficial processing variance, this phenomenon must be critically evaluated through the interplay of SME and continuous intra-granular compact consolidation. In the continuous twin-screw wet granulation line, the raw powder mass is subjected to localized, high-intensity shear stresses and compressive forces within the dynamic screw transport and kneading elements. This continuous input of mechanical energy drives efficient liquid binder distribution but concurrently forces the primary particles into a highly consolidated structural network, fundamentally reducing the internal intra-granular porosity and expanding the granule density. Consequently, when these densified granules are compressed into tablets under identical consolidation forces (3 kN), they form a tightly packed matrix that inherently restricts the capillary action required for rapid liquid ingress. This restricted water penetration directly underpins the relative retardation observed in disintegration kinetics. Crucially, despite this process-induced granule densification, our finalized formulation strategy—which locked the binder content at a micro-optimized 2.86%—successfully preempted a catastrophic collapse of drug release, maintaining high initial dissolution rates (72.1% at 10 min) and securing robust similarity factors (*f*_2_ > 50) against the reference drug (Figure 10). This confirms that while the physical disintegration pathway was delayed by the continuous engineering environment, the geometric matrix design of the formulation possessed sufficient robust buffering capacity to ensure equivalent in vitro biopharmaceutical outcomes. Beyond these microstructural mechanics, the macro-level transition to a CM platform yields decisive operational and techno-economic advantages over conventional batch processing. As validated by our preliminary process efficiency matrix, the integrated CM line achieved a dramatic lead time reduction of over 80% (compressing a 10 h sequence down to 2 h) and minimized total manufacturing costs by approximately 50% [22]. Crucially, the high level of engineering automation and system integration significantly minimized manual material handling, thereby leading to a substantial reduction in labor requirements and personnel expenses. By eliminating non-productive inter-process lag times and offline quality control approval queues, this platform showcases immense potential as a next-generation standard for smart pharmaceutical manufacturing. Nevertheless, we acknowledge that the current study possesses certain systemic boundaries that warrant further investigation. While the formulation’s buffering capacity has been established under isolated processing conditions, the dynamic state of control and real-time fluctuations during extended continuous runs have not been fully modeled. To overcome these limitations, our subsequent research will focus on two major areas: (1) implementing inline PAT—specifically embedding near-infrared (NIR) and Raman spectroscopy probes at the screw discharge and final blending chute—to verify real-time content uniformity and moisture kinetics under a Real-Time Release Testing (RTRT) framework; and (2) expanding the current design space via multivariate statistical modeling to map out the long-term operational resilience of the automated diversion loops against subtle raw material variability.

### 3.10. Stability Assessment Results

In this study, a comparative stability test was conducted for 6 months under RT and AC storage conditions to investigate the impact of manufacturing process differences on the long-term quality stability of amiodarone HCl tablets. The analysis focused on assay (specification: 90.0–110.0%) and related substances (individual unknown impurity ≤ 0.2%, impurity D ≤ 0.5%, and total impurities ≤ 1.0%). After the 6-month storage period, the average assay values for the batch and continuous processes were calculated as 97.07 ± 2.8% and 96.30 ± 2.3%, respectively, both of which successfully satisfied the predefined criteria. Furthermore, all impurity parameters, including total related substances, remained at low levels within the specified limits throughout the study period, thereby demonstrating robust stability (Table 15).

In conclusion, it was successfully confirmed that the implementation of a CM process provides quality stability comparable to the conventional batch process. Moreover, these results suggest that the continuous process may offer even further improved quality stability through future process optimization.

## 4. Conclusions

This study conducted systematic preformulation and formulation optimization research with the goal of transitioning the manufacturing process of amiodarone HCl tablets—an anti-arrhythmic agent—from conventional batch processing to innovative continuous manufacturing.

First, through AI-based formulation design programs and preformulation studies, two critical pharmaceutical challenges were identified: the low solubility and poor flowability of the API. The solubility issue was mitigated by the incorporation of Tween 80, a surfactant, which provided a baseline for evaluating equivalence with the reference drug. Simultaneously, the poor flowability was successfully improved through the application of wet granulation and the use of glidants, establishing a necessary foundation for continuous processing. Building upon these foundational studies, systematic formulation optimization was performed to overcome the initial dissolution delay reported in preceding research during the implementation of CM. By precisely modulating the binder ratio and the amount of granulation liquid, a final optimized formulation was derived, utilizing 2.86% binder and 25 mg of granulation liquid. Evaluation of this optimized formulation within the continuous process confirmed that it achieved dissolution profiles and physical properties equivalent to those of the conventional batch process, thereby successfully validating the quality equivalence between the two manufacturing methodologies.

In conclusion, this study successfully establishes an optimized continuous twin-screw wet granulation platform for amiodarone HCl tablets, demonstrating distinct scientific significance, novelty, and industrial added value. Scientifically, this work elucidates the ‘formulation buffering capacity’, proving that micro-adjustments of binder-to-liquid equilibrium (2.86% binder and 25 mg liquid) can systematically neutralize process-induced granule densification. The core novelty stems from achieving a seamless batch-to-continuous migration without altering the qualitative excipient composition of the legacy batch formulation. Beyond microstructural mechanics, this direct-transfer strategy offers immense techno-economic added value. The integrated continuous line dramatically reduces manufacturing time and production costs, while the high level of system automation eliminates manual material handling, thereby leading to a significant reduction in labor requirements and personnel expenses. These comprehensive operational advantages firmly validate this platform as a highly sustainable, next-generation smart manufacturing technology.

Nevertheless, a current limitation of this study is that the transient dynamics and real-time process monitoring during long-term continuous runs have not been fully modeled. To complement these boundaries, our future research will focus on: (1) implementing inline PAT, specifically embedding NIR and Raman spectroscopy probes to ensure RTRT, and (2) expanding the multivariate design space to optimize automated diversion loops against subtle raw material variability. Consequently, this study serves as a pivotal technological precedent for the cost-effective, regulatory-compliant modernization of low-solubility API manufacturing lines.

## Figures and Tables

**Table 1:** Composition matrix of experimental batches for amiodarone HCl tablets.

Function	Ingredient	Formulation	
		Mg/Tablet	*w*/*w* (%)
API	Amiodarone HCl	200	57.14
Filler	Starch 1500	Confidential	
Filler	Lactose	38%/27%/16%	
Binder	Povidone K25	5.71%/2.86%/2.00%/1.43%	
Solution	Purified water	50 mg/25 mg/20 mg	
Glidant	Colloidal silicon dioxide	Confidential	
Lubricant	Magnesium stearate	Confidential	
Total weight		350	100

**Table 2:** Particle size of lactose monohydrate grades.

Product	D_10_ (μm)	D_50_ (μm)	D_90_ (μm)
Tablettose 80	36	135	317
SuperTab 11SD	50	120	220
Pharmatose 200M	5	40	120

**Table 3:** Key physicochemical parameters of amiodarone hydrochloride.

Amiodarone Hydrochloride		
**CAS No.**	19774-82-4	
**Chemical formula**	C_25_H_30_Cl_2_NO_3_	
**Chemical name**	(2-Butylbenzofuran-3-yl)(4-(2-(diethylamino)ethoxy)-3,5 diiodophenyl)methanonehydrochloride	
**Molecular weight**	681.77 g/mol	
**State**	Solid	
**pKa**	pKa (strongest basic) 9.08	
**l** **og P**	7.24 (ALOGPS) 7.64 (Chemaxon)	
**l** **og S**	−5.1 (ALOGPS)	
**BCS Classification**	BCS Class II	
**Water solubility**	0.00476 mg/mL (predicted)	
**Melting point**	158∼162 °C	
**Boiling point**	635.1 °C	
**Mechanism**	As a Class III antiarrhythmic drug, it suppresses adrenergic stimulation, affects sodium/potassium/calcium channels, prolongs the action potential and refractory period in myocardial tissue, and reduces AV conduction and sinus node function.	
**Pharmacokinetics** **Parameter** **s**	Absorption	Slow and variable
	Distribution	Volume of distribution: 66 L/kg T_max_: 3–7 h
	Metabolism	Converted to the active metabolite N-desethylamiodarone by CYP2C8 and 3A4 in the liver
	Excretion	t_1/2_: 58 days Mainly cleared by fecal, kidney

**Table 4:** Accuracy assessment of the AI prediction model: a comparison of predicted and experimental values.

No.	Indicators (Unit)	AI Prediction Data	Actual Experimental Data	Match (%)
1	pH mass solubility	Figure 2	Measured value *	N/D
2	Water solubility (g/L)	0.01	0.00476	47.60
3	pH log D	Figure 2	N/D	N/D
4	log P	7.36	7.24	98.34
5	pKa	12.16	9.08	66.08
6	pKb	Null	Null	100
7	Molecular weight (g/mol)	681.78	681.77	100
8	Boiling point (°C)	115.18	635.1	18.14
9	Melting point (°C)	60.43	156	38.74
10	Bioavailability	Orally non-bioavailable, 0	0	100
11	Caco-2 permeability	Permeable with P_app_ > 8 × 10^−6^ (cm/s), 0.62	0.66	93.94
12	Lipinski’s Rule of 5	No	No	100
Average (%)				76.3

* Measured value: The detailed experimental results for pH mass solubility are presented later in Table 6.

**Table 5:** Particle size analysis results of amiodarone hydrochloride.

No.	API	PDI	D_10_ (μm)	D_50_ (μm)	D_90_ (μm)
1	Amiodarone hydrochloride	5.159	20.4	25.0	30.5
2	Amiodarone hydrochloride	4.261	19.8	24.5	30.2
3	Amiodarone hydrochloride	4.868	20.7	25.6	31.2
Average		4.763	20.3	25.0	30.6

**Table 6:** Solubility profile of amiodarone in various solvents.

No.	Solvent	Solubility of Amiodarone Hydrochloride			
		Apparent Solubility *	Equilibrium Solubility (mg/mL)		
			2 h	12 h	24 h
1	Water	++	0.11	0.16	0.16
2	Ethanol	++++	10.16	10.73	10.90
3	Methanol	+++++	73.90	73.42	79.14
4	Acetonitrile	+++	1.65	1.90	2.23
5	pH 1.2	+	0	0	0
6	pH 2.0	+	0	0	0
7	pH 3.0	+	0	0	0
8	pH 4.0	+++	1.18	1.37	3.06
9	pH 5.0	+	0	0	0
10	pH 6.0	+	0	0	0
11	pH 6.8	+	0	0	0
12	pH 7.0	+	0	0	0
13	pH 8.0	+	0	0	0
14	pH 9.0	+	0	0	0
15	pH 10.0	+	0	0	0
16	pH 11.0	+	0	0	0
17	pH 12.0	+	0	0	0
18	Water + SLS 0.1%	++	0.05	0.25	0.15
19	Water + SLS 0.5%	+++	0.40	1.02	1.18
20	Water + Tween 80 0.5%	+++	1.64	1.76	1.94
21	Water + Tween 80 1.0%	+++	2.73	3.04	3.18
22	Water + Tween 80 2.0%	+++	5.17	5.50	5.71
23	pH 4.0 + SLS 0.1%	+++	1.22	1.25	1.20
24	pH 4.0 + SLS 0.5%	+++	2.02	1.90	1.85
25	pH 4.0 + Tween 80 0.5%	+++	5.74	5.13	5.21
26	pH 4.0 + Tween 80 1.0%	+++	6.54	6.91	6.71
27	pH 4.0 + Tween 80 2.0%	+++	8.70	8.06	8.78

* +: Practically insoluble, ++: Very slightly soluble, +++: Slightly soluble, ++++: Sparingly soluble, +++++: Soluble.

**Table 7:** Stability profile of amiodarone hydrochloride in mixtures with various excipients.

No.	Compatibility Sample	Storage Condition *	Total Impurities (%)					
			Initial		2w		4w	
			Impurity D	Total	Impurity D	Total	Impurity D	Total
1	Amiodarone hydrochloride	RT	0.03	0.22	0.02	0.12	0.01	0.13
		AC			0.08	0.22	0.08	0.18
2	API: Microcrystalline Cellulose 101	RT	0.02	0.11	0.02	0.12	0.02	0.13
		AC			0.10	0.24	0.21	0.31
3	API: Lactose monohydrate 200	RT	0.02	0.12	0.02	0.12	0.02	0.12
		AC			0.09	0.21	0.08	0.19
4	API: Mannitol 200SD	RT	0.02	0.12	0.03	0.1	0.02	0.14
		AC			0.08	0.18	0.10	0.19
5	API: Pregelatinized Starch 1500	RT	0.02	0.14	0.03	0.13	0.02	0.12
		AC			0.10	0.22	0.07	0.17
6	API: Silicified Microcrystalline Cellulose 90	RT	0.02	0.13	0.03	0.13	0.02	0.13
		AC			0.09	0.19	0.11	0.18
7	API: Corn starch	RT	0.02	0.12	0.03	0.14	0.02	0.14
		AC			0.12	0.23	0.11	0.21
8	API: Croscarmellose Sodium	RT	0.02	0.11	0.02	0.07	0.03	0.09
		AC			0.07	0.13	0.09	0.17
9	API: Sodium Starch Glycolate	RT	0.06	0.20	0.07	0.15	0.06	0.16
		AC			0.18	0.26	0.23	0.30
10	API: Povidone K90	RT	0.03	0.13	0.04	0.16	0.03	0.14
		AC			0.11	0.22	0.15	0.26
11	API: Povidone K25	RT	0.02	0.13	0.03	0.14	0.02	0.13
		AC			0.09	0.21	0.15	0.26
12	API: Hydroxypropyl cellulose	RT	0.03	0.14	0.05	0.16	0.03	0.13
		AC			0.15	0.27	0.19	0.28
13	API: Hydroxypropyl Methylcellulose 2910	RT	0.03	0.14	0.05	0.17	0.03	0.13
		AC			0.11	0.24	0.10	0.24
14	API: Low Hydroxypropyl cellulose	RT	0.04	0.14	0.06	0.16	0.03	0.15
		AC			0.16	0.27	0.18	0.29
15	API: Copovidone VA64	RT	0.04	0.14	0.05	0.16	0.03	0.15
		AC			0.11	0.23	0.16	0.27
16	API: Magnesium Stearate	RT	0.10	0.20	0.12	0.20	0.14	0.26
		AC			0.28	0.39	0.35	0.50
17	API: Colloidal Silicon Dioxide	RT	0.02	0.12	0.06	0.17	0.04	0.15
		AC			0.12	0.22	0.10	0.20
18	API: Sodium Stearyl Fumarate	RT	0.25	0.35	0.29	0.40	0.38	0.39
		AC			0.51	0.62	0.43	0.53
19	API: Citric acid	RT	0	0.10	0	0.12	0	0.12
		AC			0	0.12	0	0.13
20	API: Salicylic acid	RT	0	0.24	0	0.39	0	0.11
		AC			0	0.36	0	0.11
21	API: Magnesium Carbonate	RT	0.37	0.44	1.68	1.97	5.23	6.29
		AC			1.65	1.92	5.47	6.23
22	API: Calcium Hydroxide	RT	0.32	0.37	1.24	1.48	4.62	5.48
		AC			1.32	1.58	6.20	6.56

* Storage condition: RT, long-term storage conditions (25 ± 2 °C/60 ± 5% RH); AC, accelerated conditions (40 ± 2 °C/75 ± 5% RH).

**Table 8:** Flowability assessment results of amiodarone hydrochloride.

No.	Indicators	Value	Flow Character *
1	Bulk density (g/mL)	0.4950	-
2	Tapped density (g/mL)	0.7071	-
3	Carr’s index (%)	30.00	Poor
4	Hausner ratio	1.43	Poor

* Flow character: Excellent (CI: Below 10, HR: 1.00~1.11), Good (CI: 11~15, HR: 1.12~1.18), Fair (CI: 16~20, HR: 1.19~1.25), Passable (CI: 21~25, HR: 1.26~1.34), Poor (CI: 26~31, HR: 1.35~1.45), Very poor (CI: 32~37, HR: 1.46~1.59), Very very poor (CI: Above 38, HR: Above 1.60).

**Table 9:** In-process control results for test formulations with varying binder ratios.

Tablet Properties	PVP K25 5.71%	PVP K25 2.86%	PVP K25 2.00%	PVP K25 1.43%
Hardness (kp)	6.3 ± 0.4	7.0 ± 0.5	7.3 ± 0.4	7.3 ± 0.1
Friability (%)	0.29 ± 0.2	0.29 ± 0.1	0.39 ± 0.1	0.58 ± 0.2
Disintegration (min)	7.45 ± 0.4	3.43 ± 0.4	5.18 ± 0.7	3.45 ± 0.2

**Table 10:** In-process control results for test formulations with varying granulation liquid ratios.

Tablet Properties	DW 50 mg	DW 25 mg	DW 20 mg
Hardness (kp)	7.0 ± 0.5	6.8 ± 0.4	7.1 ± 0.2
Friability (%)	0.29 ± 0.1	0.29 ± 0.1	0.54 ± 0.2
Disintegration (min)	3.43 ± 0.4	1.66 ± 0.2	1.43 ± 0.1

**Table 11:** In-process control results for test formulations with varying filler types.

Tablet Properties	Pharmatose 200M	SuperTab 11SD	Tablettose 80
Hardness (kp)	6.8 ± 0.4	6.9 ± 0.4	6.8 ± 0.3
Friability (%)	0.29 ± 0.1	0.47 ± 0.3	0.45 ± 0.2
Disintegration (min)	1.66 ± 0.2	3.67 ± 0.4	2.55 ± 0.3

**Table 12:** In-process control results for test formulations with varying filler ratios.

Tablet Properties	Pharmatose 200M 38%	Pharmatose 200M 27%	Pharmatose 200M 16%
Hardness (kp)	7.0 ± 0.3	6.8 ± 0.4	7.2 ± 0.2
Friability (%)	0.77 ± 0.1	0.29 ± 0.1	0.32 ± 0.1
Disintegration (min)	22.74 ± 0.8	1.66 ± 0.2	2.05 ± 0.2

**Table 13:** Calculated f_2_ similarity factors for comparative dissolution profiles of each experimental run.

Formulation Variable		pH4.0 *f*_2_ Value	pH1.2 *F*_2_ Value	Figure Reference
Binder ratio	PVP K25 5.71%	49.8	48.3	Figure 6
	PVP K25 2.86%	58.2	61.4	
	PVP K25 2.00%	52.4	54.6	
	PVP K25 1.43%	46.8	57.9	
Binding solution ratio	DW 50 mg	58.2	61.4	Figure 7
	DW 25 mg	70.9	54.4	
	DW 20 mg	68.3	56.1	
Filler type	Pharmatose 200M	70.9	54.4	Figure 8
	SuperTab 11SD	58.0	57.1	
	Tablettose 80	55.9	56.0	
Filler ratio	Pharmatose 200M 38%	94.0	17.1	Figure 9
	Pharmatose 200M 27%	70.9	54.4	
	Pharmatose 200M 16%	63.5	56.8	

**Table 14:** Comparison of in-process control results for tablets manufactured by batch and continuous processes.

Tablet Properties	Batch Process	Continuous Process
Bulk Density (mg/mL)	0.6500	0.6512
Tapped Density (mg/mL)	0.7200	0.8288
Carr’s Index (%)	9.7	21.4
Hausner’s Ratio	1.11	1.27
Flow Character	Excellent	Passable
Hardness (kp)	6.8 ± 0.4	7.2 ± 0.2
Friability (%)	0.29 ± 0.1	0.25 ± 0.2
Disintegration (min)	1.66 ± 0.2	6.41 ± 1.9
pH4.0 *f*_2_ value	70.9	85.2
pH1.2 *f*_2_ value	78.1	67.5

**Table 15:** Stability assessment of continuous and batch drugs after 6 months of storage.

Amiodarone Hydrochloride					
Sample	Storage Condition *	Time	Assay (%)	Impurity D (%)	Total Impurity (%)
Continuous tablet	-	Initial	94.4 ± 0.8	0.06	0.15
	RT	1M	95.5 ± 0.5	0.06	0.14
		3M	98.6 ± 3.8	0.07	0.20
		3M	98.6 ± 1.5	0.07	0.21
	AC	1M	93.4 ± 0.5	0.20	0.28
		3M	94.9 ± 0.6	0.32	0.45
		6M	98.7 ± 1.1	0.45	0.58
Batch tablet	-	Initial	97.7 ± 0.6	0.05	0.14
	RT	1M	94.2 ± 0.8	0.05	0.12
		3M	102.4 ± 2.7	0.06	0.18
		6M	97.7 ± 1.3	0.07	0.20
	AC	1M	94.6 ± 0.1	0.20	0.26
		3M	95.4 ± 1.9	0.35	0.46
		6M	97.5 ± 0.8	0.46	0.59

* Storage condition: RT, long-term storage conditions (25 ± 2 °C/60 ± 5% RH); AC, accelerated conditions (40 ± 2 °C/75 ± 5% RH).

## Data Availability

Data are available from the corresponding author upon reasonable request.

## Abbreviations

HCl, hydrochloride; CM, continuous manufacturing; PAT, process analytical technology; QbD, Quality by Design; API, PVP K25, povidone K25; active pharmaceutical ingredient; AI, artificial intelligence; SMILES, Simplified Molecular Input Line Entry System; GUI, graphical user interface; DSC, differential scanning calorimetry; DLS, dynamic light scattering; PMMA, disposable polymethyl methacrylate; PDI, polydispersity index; USP, United States Pharmacopeia; RC, regenerated cellulose; HPLC, high-performance liquid chromatography; RT, room temperature; RH, relative humidity; AC, accelerated conditions; CI, Carr’s index; HR, Hausner ratio; IPC, in-process control; kp, kiloponds; LOD, loss on drying; HDPE, high-density polyethylene; BCS, biopharmaceutics classification system; Tween 80, polysorbate 80; UV-Vis, ultraviolet/visible; CQA, critical quality attribute; SLS, sodium lauryl sulfate; f_2_, similarity factor; CPP, critical process parameter; SME, specific mechanical energy; RTD, residence time distribution; NIR, near-infrared; RTRT, Real-Time Release Testing
